# The effect of value on context and target recollection in memory for truth and falsity

**DOI:** 10.3758/s13421-024-01554-3

**Published:** 2024-04-03

**Authors:** Daria Ford, Marek Nieznański

**Affiliations:** 1 Institute of Psychology, Cardinal Stefan Wyszyński University in Warsaw, Warsaw, Poland; 2 Department of Psychology, School of Social Sciences, University of Mannheim, Mannheim, Germany

**Keywords:** Memory for truth, Value-directed remembering, Context memory, Dual-recollection theory, Prioritization

## Abstract

Memory for truth and falsity has recently been investigated from the perspective of the dual-recollection theory, showing better context and target recollection for truth than falsity. In this paper, we examine whether these memory effects obtained for true statements are similar to the value effect, whereby true statements are given higher priority in encoding. For this purpose, we implemented value-directed remembering (VDR) into the conjoint-recognition paradigm. In our first experiment, the primary goal was to verify how VDR influences the processes defined by dual-recollection theory. At study, prioritized/important items were linked to higher numerical values (e.g., 10), while unimportant ones had lower values (e.g., 1). At test, the participants’ task was to recognize whether a particular sentence was important, unimportant, or new. We found that both context and target recollection were better for important items. In the second experiment, the main goal was to study the combined effects of importance and veracity on memory. In the between-subjects design, participants were monetarily rewarded for memorizing true or false sentences. The results demonstrated differences in the ability to prioritize truth over falsity. Specifically, we found a substantial increase in context recollection for prioritized true information but not for prioritized false information. Moreover, we found higher context recollection for true than false sentences in the true-prioritized condition, but not in the false-prioritized condition. These results indicated that people are able to prioritize true information better than false, and suggested that memory for truth may be a special case of the value effect.

## Introduction

In the modern world, people are faced with an increasing amount of information to process, some of which is true and some false. Prioritization of the incoming information has a key importance on its memorization, so evaluation and selection of valuable information seems to be critical for memorization from a practical perspective. The key question is how we prioritize true and false information and whether we are capable of prioritizing these two types of information in the same way. In this article, we also explore whether value-directed remembering can partly explain better recollection of truth than falsity, since true information may at the same time be more valuable to subjects. To investigate whether the truth effect could be an instance of the value effect, we examine the similarities and differences in the studied effects at the level of the basic memory processes postulated in the dual-recollection theory (Brainerd et al., 2014, 2015).

In the academic debate on memory for truth and falsity, most researchers have focused their efforts on determining whether a model that assumes symmetrical processing of truth and falsity (the so-called Cartesian model) or a model that assumes differences in the stages of encoding truth and falsity (the Spinozan model) provides a better theoretical account (e.g., Ford & Nieznański, 2023; Gilbert et al., 1990; Nadarevic & Erdfelder, 2013, 2019; Street & Kingstone, 2017). In our previous experiments on this issue, we found that true information was remembered better than false information (Ford & Nieznański, 2023; Niedziałkowska & Nieznański, 2021). This paper aims to further investigate the nature of processing truth and falsity by introducing the procedure of value-directed remembering into the memory for truth and falsity task.

The processes underlying performance in the truth/falsity memory tasks were analysed from different theoretical perspectives in the literature. Research referring to the source-monitoring framework (Johnson et al., 1993) that assumed a two-high threshold model of memory recognition (Bayen et al., 1996) generally indicated that processing of feedback information does not differ for “true” and “false” feedback (Nadarevic & Erdfelder, 2013, 2019, Experiment 1), except in one case (Nadarevic & Erdfelder, 2019, Experiment 2, control group), where detection for true information was significantly better than for false information. Another perspective is offered by a one-high threshold model based on dual-recollection theory (Brainerd et al., 2014, 2015), which has indicated that recollection is enhanced for “true” feedback information (Ford & Nieznański, 2023; Niedziałkowska & Nieznański, 2021).

Dual-recollection theory refers to the distinction between recollection and familiarity from dual-process theories (Yonelinas, 2002), but it introduces recollection as a bivariate process, comprising target recollection and context recollection (Brainerd et al., 2014, 2015). Target recollection refers to a conscious reinstatement of events related to the target per se, and context recollection is a conscious reinstatement of contextual elements that were present during the target events. Familiarity is an automatic process, which refers to general activation of a memory trace evoked by the presented stimulus without recollecting the previous event (Yonelinas, 2002). In our research on truth/falsity memory, we found that context recollection for true statements was better than for false sentences (Ford & Nieznański, 2023; Niedziałkowska & Nieznański, 2021). This effect was not observed only when the refreshing maintenance process was disrupted during encoding (Ford & Nieznański, 2023). Furthermore, target recollection was also better for true in comparison to false sentences under full-attention condition (Ford & Nieznański, 2023) or in the specific condition, when participants’ pre-experimental knowledge was consistent with the feedback received during study phase of the memory experiment (Niedziałkowska & Nieznański, 2021, Experiment 2).

Another factor that possibly enhances recollection is prioritization. Enhancing people’s ability to prioritize learning an important or perceived-as-valuable piece of information over others can be achieved via different types of procedure, and typically, cue-based and reward-based prioritization procedures (Jeanneret et al., 2023). Value-directed remembering (VDR) is a reward-based procedure in which a task consists of items to be remembered along with varying degrees of value (e.g., ranging from 1 to 10). Participants are asked to prioritize memorizing higher value (10) items over lower value (1) items. Previous research has shown that adding a numerical value to memorized items influences memory performance for items themselves, leading to better memory performance in a high-value condition (e.g., Castel et al., 2002; Hennessee et al., 2017; Villaseñor et al., 2021), whereas in some research the effect of value was not limited to the item only, but also enhanced memory for the context (source) in which the item was presented, for color (Yin et al., 2021) or location (Cohen et al., 2019). Furthermore, in the studies on value effect using the Remember/Know paradigm, value enhanced “Remember” responses, which rely on a recollection rather than familiarity process (Elliott & Brewer, 2019; Hennessee et al., 2017, 2018). Therefore, in studies on value effect in the dual-process theories, it is particularly relevant to focus on the contribution of processes related to recollection.

As dual-recollection theory distinguishes two types of recollection, it is interesting to see how value might impact the contribution of context and target recollection. One of the possible explanations of the value effect is that more attentional resources are allocated during the study phase to high-value items (Allen, 2019). According to Knowlton and Castel (2022), the effects of VDR can be both automatic and strategic, so the process of selecting important information occurs on the conscious as well as the subconscious/neurobiological level. Therefore, it is possible that people unconsciously allocate attention to the more useful and thus valuable information. We showed that context recollection for true sentences (but not for false sentences) was significantly decreased due to cognitive load (Ford & Nieznański, 2023), which suggests that attentional (processing) resources are allocated to true (more than false) information.

The distinctive processing of both truthfulness and importance feedback, leading to better recollection, may be due to the conceptual nature of these cues and their higher utility. As Rahhal et al. (2002) demonstrated, in comparison with traditional perceptual source cues, age-related deficits in source memory are attenuated when conceptual source cues are used. It is possible that higher utility of conceptual source information enhances binding with item information, even in older adults. Note that conceptual cues in comparison with perceptual cues are probably processed on a deeper level, as suggested by a classic levels-of-processing approach (Craik & Lockhart, 1972). Moreover, it seems that the most valuable or useful information people can render immune to the effects of divided-attention (Middlebrooks et al., 2017). Arguably, this ability to strategically allocate resources to valuable information is the basis for the metacognitive assumption that if we have forgotten something, it must be less valuable than something we remember (Castel et al. 2012; Rhodes et al., 2016).

Moreover, highly valued items are processed on a deeper semantic level (Cohen et al. 2017), and our recent experiment (Ford & Nieznański, 2023) has shown that in the condition where deep-semantic processing was impaired (by blocking refreshing), the effect of higher context recollection for truth was not observed. Since impairment of deep (elaborative) processing eliminates the advantage of truth memory over false memory, then deep processing may play a greater role in remembering truth than falsity. Research in the dual-recollection model indicated that context recollection is sensitive to the level of processing (Nieznański, 2020), thus deep processing of truth information may be reflected in an increase of this particular process. Naturally, deep (elaborative) processing requires more attention (Craik & Lockhart, 1972), so these two factors are closely related or even overlap.

We hypothesize that context and target recollection are enhanced for true information because people focus their attention (or elaborate) on it for the purpose of incorporating this information into their general knowledge. In other words, people may automatically regard true information as more valuable and useful than false information, and in this way prioritize it. The current article aimed to investigate if prioritizing by assigning high/low value to the memorized items affects the processes of dual-recollection theory similarly to assigning information on the veracity status to an item. In order to do that, we first validated how value impacts the processes of dual-recollection theory and compared the value effect and the true/false information effect that we observed in our previous studies (Ford & Nieznański, 2023; Niedziałkowska & Nieznański, 2021). In the second experiment, we introduced value (high/low) in the memory for truth and falsity task, to see if assigning a value influences memory processes in the same way for truth and falsity. Our hypothesis was that indicating that something is true is equivalent to assigning it a higher value, and that indicating that it is false is the same as assigning it a low value. Moreover, if false information is considered unimportant by default and its encoding is inhibited, prioritizing it (by assigning it a high value in the study phase of an experiment) introduces inconsistency, and as a result of this inconsistency, prioritization may be ineffective. Therefore, we do not expect any enhancement of memory performance for false-prioritized information. Prioritizing the truth may, on the one hand, amplify its value, or, on the other hand, make no change if the importance was already maximal. The additive effect seems to be more probable when hypothetical mechanisms of value effect and truth effect are at least partially distinct – for example, if one is based on attention allocation and the other on elaboration or deep-level processing.

We conducted two experiments in order to see what impact value has on memory processes defined in the dual-recollection theory (Experiment 1) and we also manipulated value to see if people are able to prioritize memorization of true sentences in the same way as false sentences (Experiment 2).

## Experiment 1

The main aim of this experiment was to study VDR in the dual-recollection model and to see the contribution of the latent processes of the model when prioritization is introduced. We hypothesized that prioritization will mostly influence recollection, which is a consciously controlled memory process. This is supported by results from studies using remember/know procedures that indicated that proportions of “remember” responses are higher for items marked as important than as unimportant (Cohen et al., 2017; Elliott & Brewer, 2019; Hennessee et al., 2017). This boost in item remembering should be reflected in an increased target recollection parameter of the dual-recollection model. Furthermore, we wanted to see if the context recollection parameter for important sentences would be better than for unimportant ones, because in some research (Cohen et al., 2019; Yin et al., 2021) memory for context differed between high-value and low-value items, and in other research there was no statistically confirmed difference (Hennessee et al., 2017).

### Methods

#### Participants

Forty-eight students (30 female, *M*_*age*_ = 23.21 years, *SD* = 4.39, range 19–41 years) received a 35PLN (9$) shop voucher for participating in the study, of which the half with higher scores received a reward – an additional 35PLN voucher. The study was built in *OpenSesame* (Mathôt et al., 2011), and, due to the COVID-19 pandemic, conducted online on the Mindprobe platform. The participants were tested individually and instructions were presented on a computer screen. It was obligatory for the participants to open the study on a computer device and not on a smartphone or an iPad.

We computed a priori power analysis using G*Power (Faul et al., 2007). To detect a medium-sized (or smaller) effect (*d* ≤ 0.5), with the power 1 – β = .80. and α = .05 (two-tailed) on the difference in the rate of accurate identifications between important and unimportant sentences, it was necessary to recruit at least 34 participants. Participants volunteered to take part in the study, and since we did not want to reject any volunteers, this resulted in a larger sample size than indicated in the power analysis.

#### Materials and procedure

We used 80 trivia statements adapted from Nadarevic and Erdfelder (2013) (e.g., “The biggest aggregation of desert salt is in Iran.”; “Depending on the season, oysters can change sex.”; and “The electronic index of the trading market is called Xetra.”; for details of the adaptation procedure, see Niedziałkowska & Nieznański, 2021), some of which were true and some false. The selected sentences were of neutral truth value to the participants, meaning they were unsure if sentences are rather true or false as in our earlier studies (Ford & Nieznański, 2023; Niedziałkowska & Nieznański, 2021) and the studies of Nadarevic and Erdfelder (2013, 2019). The information on truth/falsity of the statements was not shown to the participants in this experiment. We chose trivia sentences as material to keep the consistency between Experiment 1 and Experiment 2 (in which veracity status is an independent variable). In this experiment, 72 sentences were targets or distractors and eight sentences were used as recency or primacy buffers. The assignment of sentences to targets or distractors, the context at study (important/unimportant), and the type of test probe were counterbalanced across the participants, resulting in 12 unique lists. This means that one sentence could appear in one of 12 possible combinations. These versions were distributed equally among participants. At study, the participants were asked to remember 44 trivia sentences along with their important/unimportant status (36 targets, four buffer sentences at the beginning, and four at the end of the learning phase). At test, another 36 sentences were mixed with targets as distractors.

Participants were instructed to remember sentences during the test and informed that for remembering important sentences they will receive 10 points, and for unimportant sentences 1 point. They were also notified that the top half of participants would later on receive an additional bonus (35 PLN shop voucher). We applied a binary high-low distinction between items adapted from Yin et al. (2021). This format helps participants to easily distinguish between high-low value items and focus on high-value items. During the study phase, the sentences were displayed in the center of the computer screen in black font (Times New Roman, 28-point size) on a white background along with the important/unimportant information, sequentially, each for 6 s. During the test phase, participants were asked three types of questions: (a) *Was this sentence presented as important*? (b) *Was this sentence presented as unimportant*? and (c) *Was this sentence presented either as important or as unimportant*? For each sentence there was one question displayed and the types of probe questions ascribed to each of the sentences were counterbalanced across the participants. During the test phase, the sentences were displayed in random order at a self-paced rate. The participants were asked to respond by pressing keys on a keyboard: key T for “yes” (in Polish *tak*) and N for “no” (in Polish *nie*). The test instructions stressed that when a sentence is new, participants should answer “no” on every type of question.

#### Design

Independent variables (feedback type: Important vs. Unimportant; and test probe type: *Important*? *Unimportant*? Or *Important or Unimportant*?) were manipulated within the participants. The dependent variables were corrected acceptance rates of participants’ responses (CAR), and the parameters of the multinomial model, which estimate the contribution of latent memory processes (context recollection, target recollection and familiarity) and guessing biases to memory task performance.

#### Measurement model

To analyze underlying memory processes contributing to truth/falsity memory task performance, we employed a method of multinomial processing tree modeling, which enables measurement of latent cognitive processes disentangled from the influence of guessing. In this research, we have used the dual-recollection model (Brainerd et al., 2015) in order to estimate the contribution of three latent memory processes underlying the prioritization effect: target recollection, context recollection, and familiarity.

**Fig. 1:**
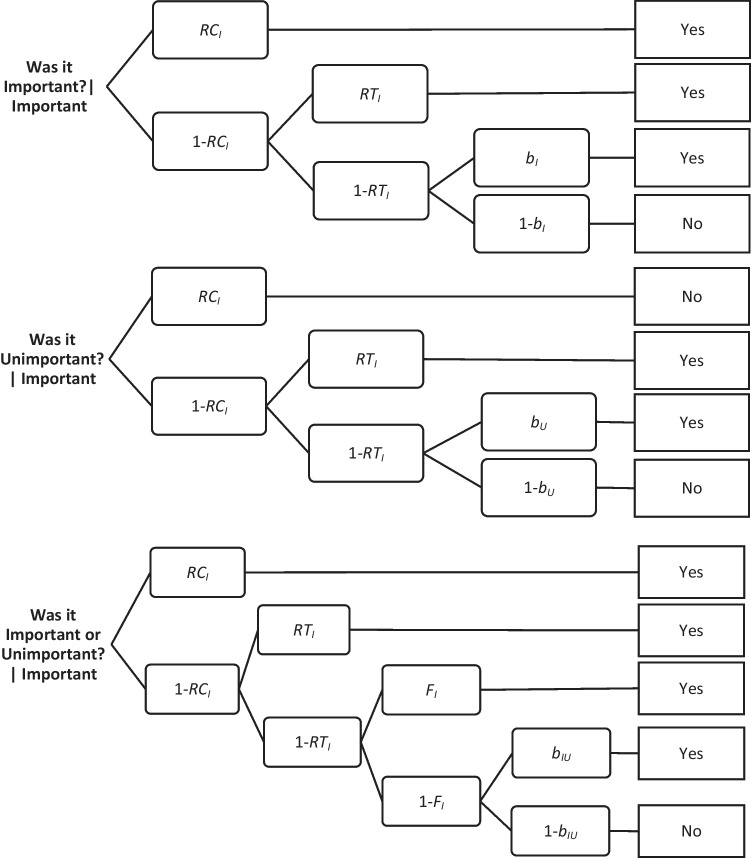
A part of the multinomial dual-recollection model for memory for important and unimportant sentences. This model is based on Brainerd et al. (2015, Fig. 1), where *RC* – the context recollection parameter, *RT* – the target recollection parameter, *F* – the familiarity parameter, *b* – the response bias parameter; where subscripts _I_ – Important, _U_ – Unimportant

In our experiment, the context was determined as the “important” or “unimportant” feedback. As depicted in Fig. 1, on the *Was it Important?* and *Was it Unimportant?* Probes for targets’ consistent contexts (*Was it Important?*|Important or *Was it Unimportant?|*Unimportant), the test items are accepted if either *RC* or *RT* is successful, and if neither is, response bias *b* can result in acceptance. On the *Was it Important?* and *Was it Unimportant?* probes for targets’ inconsistent contexts (*Was it Important?*|Unimportant; or *Was it Unimportant?|*Important), the test items are rejected if *RC* is successful, accepted if *RC* fails and *RT* is successful, and response bias *b* can result in acceptance if neither is successful. On the *Was it Important or Unimportant?* Probes for targets (*Was it Important or Unimportant?*|Important or *Was it Important or Unimportant?|*Unimportant), the test items are accepted if *RC*, *RT*, or *F* is successful, and response bias *b* can result in acceptance if all of these processes fail. The third type of probe is a question whether the item was presented at all. For the distractors, the response bias *b* parameters can produce acceptances of all three types of probes (Brainerd et al., 2015).

### Results and discussion

#### Results based on descriptive measures

**Fig. 2:**
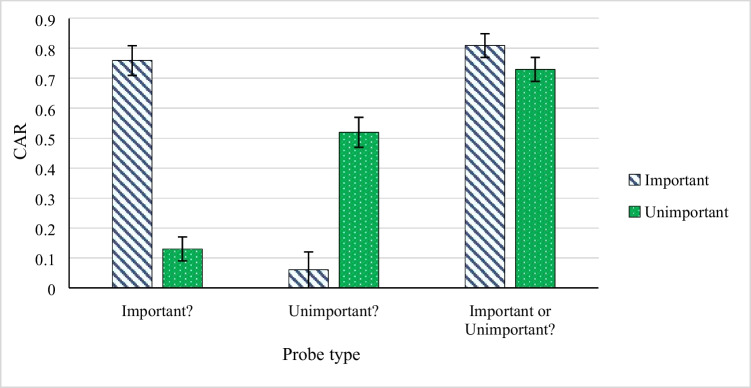
Mean (SE) corrected acceptance rates (CAR) in Experiment 1

The data, materials and code for both experiments are available at https://osf.io/9gzyc/. The corrected acceptance rates (CAR, i.e., the probability of a “yes” response for old items minus the probability of a “yes” response for distractors) depending on the test probe type are presented in Fig. 2. We conducted analyses in JASP (JASP Team, 2019), and performed a paired-samples *t*-test on CARs between the sentence types. First we compared accurate identifications of sentences’ context, that is CAR for important sentences on the “Important?” probes and CAR for the unimportant sentences on the “Unimportant?” probes, *t*(47) = 3.65, *p* < .001, indicating a medium effect size, Cohen’s* d* = 0.53. Second, we compared misidentifications of sentences, that is CAR of important sentences on the “Unimportant?” probes and CAR of unimportant sentences on the “Important?” probes, and we found no statistically significant difference here *t*(47) = 1.17, *p* = .246. Moreover, we performed a Wilcoxon signed rank test between CAR for important versus unimportant on the “Important or Unimportant?” probes, which revealed a significant difference in favor of important sentences and a medium effect size, *W* = 104.50, *p* = 0.001, *r*_rb_ = 0.63. In sum, we found that participants significantly better identified important sentences as important than unimportant as unimportant, and that they better identified target items as old when sentences were important compared to unimportant.

#### Results based on multinomial modelling

Multinomial processing tree (MPT) models account for categorical data and estimate the probability of latent cognitive processes, including guessing bias (for reviews, see Batchelder & Riefer, 1999; Erdfelder et al., 2009). In a standard approach all data are aggregated across participants for statistical reasons, but a disadvantage of this approach is that we do not account for the heterogeneity of the individual participants’ results (Heck et al., 2018). To address this limitation, we applied a Bayesian hierarchical extension of the MPT model, which is gaining increasing popularity in memory research (e.g., Kroneisen et al., 2021; Nieznański et al., 2024; Schaper et al., 2022). All analyses were conducted using the R package TreeBUGS (Heck et al., 2018), which uses the Markov-chain Monte-Carlo methods (Plummer, 2003) to fit Bayesian hierarchical MPT models. We followed the latent-trait approach (Klauer, 2010), which, as the prior distribution on a group level, uses the multivariate normal distribution of the transformed individual parameters. The results of the parameter estimates and the corresponding Bayesian Credibility Intervals (BCIs) of the dual-recollection multinomial model are presented in the Table 1. The model is based on nine independent empirical categories: 3 probe types (Important?, Unimportant?, Important or Unimportant?) × 3 test items (Important Targets, Unimportant Targets, Distractors), and nine parameters (context recollection for important sentences – *RC*_*I*_, context recollection for unimportant sentences – *RC*_*U*_, target recollection for important sentences – *RT*_*I*_, target recollection for unimportant sentences – *RT*_*U*_, familiarity for important sentences – *F*_*I*_, familiarity for unimportant sentences – *F*_*U*_, guessing yes on Important? – *b*_*I*_, guessing yes on Unimportant? – *b*_*U*_, guessing yes on Important or Unimportant? – *b*_*IU*_). Model fit was assessed with posterior-predicted *p* values and indicated a satisfactory fit for both the mean structure (*T*_*1*_: *p* = .539), and the covariance structure (*T*_*2*_: *p* = .236) of the observed individual frequencies. To test for differences in parameters between context recollection for important versus unimportant items, we sampled the parameter difference, Δ*RC* =* RC*_*I*_ – *RC*_*U*_*.* There is evidence for a difference if the BCI for the parameter difference estimate does not contain zero. For Δ*RC* = .27, BCI = [.04, .49], showing a substantial difference between context recollection parameters for important versus unimportant sentences. To test for differences in parameters between target recollection for important versus unimportant items, we sampled the parameter difference, Δ*RT* = *RT*_*I*_ – *RT*_*U*_. For Δ*RT* = .26, BCI = [.08, .43], indicating clear difference.

**Table 1:** Parameter estimates (SD) and 95% Bayesian Credibility Intervals of the dual-recollection model observed in Experiment 1 for important vs. unimportant sentences

	Parameter	
	Important	Unimportant
Context recollection	.62 (.08) [.44, .76]	.35 (.09) [.17, .51]
Target recollection	.52 (.07) [.38, .65]	.26 (.06) [.14, .38]
Familiarity	.22 (.16) [.01, .60]	.10 (.08) [.00, .30]
Guessing old (I?)	.01 (.01) [.00, .03]	
Guessing old (U?)	.05 (.03) [.01, .13]	
Guessing old (IorU?)	.02 (.01) [.00, .05]	

To test for differences in familiarity parameters between important and unimportant items, we sampled the parameter difference, Δ*F* = *FI* – *FU*. The difference was not substantial, Δ*F*= .11, BCI = [–.20, .51]. However, familiarity only occurs when the processes of recollection fail, and therefore it is located lower in the order of parameters of the dual-recollection model. For that reason, when recollection processes are at a high level, the familiarity parameter is estimated with less data, leading to a larger standard error (Brainerd et al., 2014). Due to this limitation in statistical reliability, we do not draw any conclusions in the discussion section about differences in this parameter.

There was no substantial difference in response bias (Δ*b* = *b*_*I*_ – *b*_*U*_) between guessing “yes” on Important? and guessing “yes” on Unimportant? probes Δ*b* = .04, BCI = [–.004, .12].

In sum, we found that the priority manipulation in the VDR procedure influenced both context and target recollection parameters, resulting in better memory for important in comparison with unimportant statements. The dual-recollection model was successfully applied in the VDR procedure showing the effects on recollection processes. Conscious reinstatement of target features as well as conscious reinstatement of contextual features are clearly better for important (high-value) sentences. These results are similar to the results in our previous studies (Ford & Nieznański, 2023; Niedziałkowska & Nieznański, 2021) for true versus false sentences, where context and target recollection was higher for true sentences compared with the false ones.

## Experiment 2

In the second experiment we aimed to study the influence of prioritization on memory for truth and falsity. Our main goal was to compare in one experiment the effects of prioritization and the effects of true feedback on the processes postulated in the dual-recollection theory. We aimed to clarify if the enhancement of context and target recollection for truth might be a special case of the value effect, because – as the philosophical tradition suggests – truth is a value itself. We also wanted to see if adding a numerical value to true/false information would allow people to prioritize it in the same way for true and false information. To investigate this, we wanted to see if there is a difference between true and false sentences in target and context recollection parameters within the two conditions. If people are able to prioritize true and false information in the same way, we can expect, based on the results of Experiment 1, that context and target recollection will increase for the type of information that is prioritized – true information when truth is indicated as important, and false information when false is indicated as important. However, interaction effects are also possible if prioritizing one type of information is more or less effective than prioritizing the other type. On the one hand, it is possible that false information is actively inhibited during encoding to avoid knowledge contamination, and prioritizing false information may not be sufficient to overcome this inhibition. On the other hand, the memory for true information may already be enhanced by better semantic elaboration or increased attention, and prioritizing it may affect the same mechanisms that have already worked. An additive effect would be more likely when the mechanisms leading to the enhancement of memory for truth do not overlap with those leading to better memory of important information.

### Methods

#### Participants

In this experiment, 74 undergraduate university students (66 female) were recruited (*M*_age_ = 20.42 years, *SD* = 1.07, range 18–26 years). All of them were given course credit for their participation and participants who gained most points were also rewarded with shop vouchers for 50PLN or 85PLN (12$ or 21$), depending on their performance level. The participants were randomly assigned to one of two conditions: true-prioritized (36 students) and false-prioritized (38 students). The study was conducted in the University Lab using the E-Prime 2.0 software (Psychology Software Tools, Pittsburgh, PA, USA). We ran the experiment for five participants at a time at individual stations.

A priori power analysis using G*Power (Faul et al., 2007) showed that to detect a medium (or smaller) size effect (*f* ≤ 0.25), with the power 1 – β = .80, and α = .05 for ANOVA with repeated measures and within-between interaction, we should recruit at least 34 participants. We accepted more volunteers than indicated in the power analysis to ensure all willing students had the opportunity to earn course credits.

#### Materials and procedure

We used the same 80 trivia statements as in Experiment 1. The procedure was similar to the procedure of Experiment 1 (with some minor differences described below); however, this time the context provided was information about the truth status of the sentences. We divided participants into two groups and informed participants in one group that for accurate memory for sentences which are tagged as “truth” they will get 10 points and for sentences tagged as “falsity” they will receive 1 point (true-prioritized group), while in the other group for accurate memory of sentences tagged as “falsity”, participants were informed to receive 10 points and for accurate memory for “truth” – just 1 point (false-prioritized group).

During the test phase, participants were asked three types of question: (a) *Was this sentence presented as truth*? (b) *Was this sentence presented as falsity*? and (c) *Was this sentence presented either as truth or as falsity*? The participants were informed again that in a true-prioritized group for a correct recognition of sentence marked as “truth” they will receive 10 points, for sentence tagged as “falsity” they will receive 1 point, and for a new sentence they will receive 2 points. For an incorrect recognition they will lose 2 points. In the false-prioritized group, participants were given the reversed number of points for correct recognitions of true and false sentences and the same amount for new sentences. The set of sentences in each group was divided into equal parts that were allocated to each of the three test probe types. These versions were given to an approximately equal number of participants.

#### Design

The design was a 2 (priority: true-prioritized vs. false-prioritized) × 2 (true vs false sentences) mixed design with priority being manipulated between subjects and the veracity of the sentences within subjects. The dependent variables were descriptive measures (CAR) and the parameters of the dual-recollection multinomial model, representing latent memory processes and guessing biases.

#### Measurement model

In Experiment 2 we used the same model as described in Experiment 1, but in this experiment, the context was determined by the “truth” or “falsity” feedback, because the aim was to measure the influence of VDR manipulation on memory for truth and falsity. Thus, the questions asked were *Was it Truth? Was it Falsity?* or *Was it Truth or Falsity?*

### Results and discussion

#### Results based on descriptive measures

**Fig. 3:**
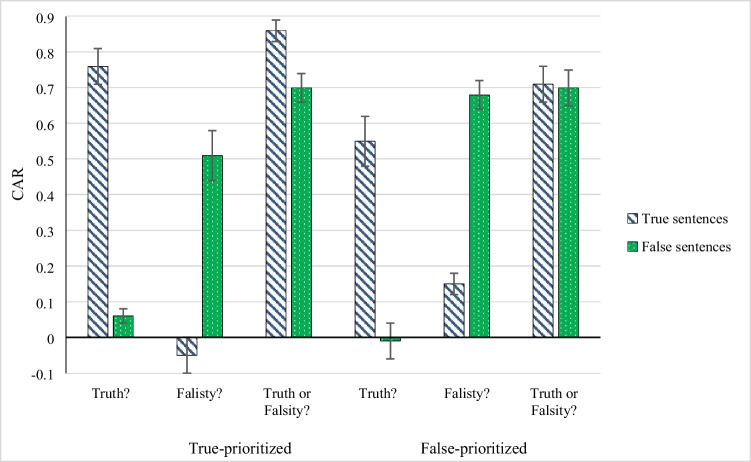
Mean (SE) corrected acceptance probabilities (CAR) in Experiment 2 for true-prioritized condition and false-prioritized condition

The corrected acceptance rates CAR are presented in Fig. 3. For the accurate mean CAR for feedback information, a 2 (true sentence identified as true vs false sentence identified as false) × 2 (true-prioritized vs. false-prioritized) mixed ANOVA was calculated, with sentence veracity manipulated within subjects, and the priority type manipulated between subjects. No main effects of sentence veracity, *F*(1, 72) = 1.47, and priority type, *F*(1, 72) = 0.06, were observed; however, a large effect of interaction was revealed, *F*(1, 72) = 15.88, *p* < .001, *η*_*p*_^*2*^ = .18. Because the ANOVA assumption of equal variances among conditions was not met, we reran this analysis with arcsine-transformed data, and we also found a significant interaction effect and non-significant main effects.

Similar results were found with mixed ANOVA for the inaccurate mean CAR for feedback information, with sentence veracity (true sentence identified as false vs. false sentence identified as true) manipulated within-subjects, and the priority manipulated between subjects. We found no main effect of veracity, *F*(1, 72) = 0.40, and priority type, *F*(1, 72) = 2.21, but a significant and large effect of interaction was revealed, *F*(1, 72) = 11.67, *p* = .001, *η*_*p*_^*2*^ = .14. And again, to double-check for artifactual results due to ANOVA assumptions violation, we analyzed the arcsine-transformed data and obtained the same conclusions to those using untransformed CARs.

Finally, we calculated mixed ANOVA for the accurate mean CAR for sentence presentation (i.e., recognition of true sentences as presented or false sentences as presented). We found a significant main effect of veracity, *F*(1, 72) = 5.51, *p* = .02, η_p_^2^ = .07, no effect of priority type, *F*(1, 72) = 2.47, and no interaction,* F*(1, 72) = 3.99. This time the ANOVA assumption of equal variances among conditions was met.

#### Results based on multinomial modelling

The results of the parameter estimates and the corresponding BCIs of the dual-recollection multinomial model are presented in Table 2. There were two models: one for the true-prioritized condition and one for the false-prioritized condition, each of them based on 12 independent empirical categories: 3 probe types × 4 test items (Truth Targets, Falsity Targets, Truth Distractors, Falsity Distractors), and nine estimated parameters. Similar to Experiment 1, we found a satisfactory model fit for true-prioritized model (*T1*: *p* = .689; *T2*: *p* = .293) and for false-prioritized model (*T1*: *p* = .511; *T2*: *p* = .229).

**Table 2:** Model parameter estimates (SD) observed in Experiment 2 and 95% Bayesian Credibility Intervals of the dual-recollection model

	Parameter			
	True-prioritized condition		False-prioritized condition	
	True sentences	False sentences	False sentences	True sentences
Context Recollection	.75 (.05) [.66, .84]	.51 (.07) [.36, .63]	.59 (.05) [.49, .68]	.50 (.07) [.35, .63]
Target Recollection	.33 (.10) [.12, .52]	.20 (.05) [.11, .30]	.30 (.07) [.16, .43]	.31 (.07) [.18, .44]
Familiarity	.35 (.21) [.02, .81]	.22 (.11) [.02, .45)	.14 (.13) [.00, .48]	.17 (.19) [.01, .43]
Guessing old (T?)	.05 (.02) [.01,.09]		.09 (.05) [.02, .20]	
Guessing old (F?)	.09 (.05) [.02, .20]		.02 (.01) [.00, .04]	
Guessing old (TorF?)	.03 (.02) [.00, .06]		.03 (.02) [.01, .07]	

When truth was prioritized, to test for differences in parameters between context recollection for true (*RC*_*T*_) vs false (*RC*_*F*_) items, we sampled the parameter difference Δ*RC* = *RC*_*T*_ – *RC*_*F*_. For Δ*RC* = .25, BCI = [.11, .41], showing a substantial difference between context recollection parameter for true versus false sentences. Difference of target recollection parameter, Δ*RT* = *RT*_*T*_ – *RT*_*F*_, was not substantial, Δ*RT*= .12, BCI = [–.10, .33]. The difference was also not substantial for familiarity parameter (Δ*F *= *F*_*T*_ – *F*_*F*_), Δ*F*= .14, BCI = [–.29, .64]. There was no difference in guessing bias (Δ*b* = *b*_*T*_ – *b*_*F*_) between guessing yes on Truth? and guessing yes on Falsity?, Δ*b* = –.04, BCI = [–.14, .04].

When falsity was prioritized, there were no differences in context recollection, Δ*RC* = –0.08, BCI = [–.26, .08], target recollection, *ΔRT* = 0.003, BCI = [–.18, .19], and familiarity, Δ*F*= .04, BCI = [–.33, .36], between true and false sentences. The only substantial difference was in guessing bias between guessing yes on Truth? And guessing yes on Falsity? Δ*b* = .07, BCI = [.001, .18], indicating that when falsity was prioritized people tended to guess that an unrecognized item is true rather than false.

Between-subjects comparisons showed that there was a difference between context recollection parameter for true sentences, *p*_*b*_ = .002, BCI = [.08, .45], but not for false sentences, *p*_*b*_ = .809, BCI = [–.25, .09]. There were no differences between conditions in target recollection parameters neither for true sentences, *p*_*b*_ = .434, BCI [–.22, .25], nor for false sentences, *p*_*b*_ = .857, BCI [–.26, .08]. Similarly, there were no differences between conditions in familiarity parameters neither for true sentences, *p*_*b*_ = .255, BCI [–.27, .67], nor for false sentences, *p*_*b*_ = .270, BCI [–.29, .38]. Additionally, no differences in the guessing bias were found.

In sum, in Experiment 2 a value-directed remembering procedure has been applied in order to measure the effect of prioritization on memory for truth and falsity. Analysis based on descriptive measures revealed interaction effects between prioritization and sentence veracity. These interaction effects occurred for correct identifications of sentence veracity and for misidentifications, but not for old/new recognitions. This means that the ability to prioritize information depends on the sentence veracity. We estimated the contribution of dual-recollection parameters for two conditions; true-prioritized and false-prioritized, showing also in multinomial analyses that people are able to effectively prioritize true information but not false.

## General discussion

The aim of this paper was to study the effects of prioritization on memory processes defined in the dual-recollection theory (Brainerd et al., 2014, 2015) and explore if there is an analogy in the effects of value and veracity on recollection. In our earlier studies (Ford & Nieznański, 2023; Niedziałkowska & Nieznański, 2021) we found better context and (under certain conditions) target recollection for true information compared to false information. Similarly, in the presented experiments, we found an enhancement of context and target recollection for important as compared to unimportant information. These results allow for some speculation as to whether the nature of the effects of veracity and value on memory are indeed alike, and if so, whether the former can be seen as a special case of the latter. From a traditional philosophical perspective, truth can be considered as a value itself, and assigning the label "truth" to the sentence can be understood as indicating that it is important or simply worthy of attention.

In the first experiment, we investigated the effect of value on the processes proposed in the dual-recollection theory. We found that prioritization enhanced both context and target recollection, which is in line with the findings of Yin et al. (2021), where for both item and context, memory was better in the high-value condition. These results suggest that high-value items are processed similarly to true sentences, whereas low-value items are processed similarly to false sentences. In order to check what the influence of prioritization is on memory for truth and falsity, we conducted a second experiment. In this experiment, there were two experimental conditions with between-subjects priority manipulation. The introduced context was information about sentence veracity. Our main goal was to examine the interaction between importance and veracity of the information. The results indicated the presence of the value effect on memory for true sentences, but no effect on memory for false sentences. Moreover, context recollection was higher for truth compared to falsity in the true-prioritized condition and between-conditions comparison for true sentences indicated higher context recollection in the true-prioritized condition than the false-prioritized condition. This shows that people can easily prioritize true information, but they lack this ability when it comes to false information. In support for these findings based on multinomial modeling, an analysis on descriptive measures also revealed an interaction effect between prioritization and veracity. That is, we found that proportions of accurate identifications are higher for true information than for false information when truth is prioritized but not when falsity is prioritized. Similarly, we found higher proportions of misidentifications of true information as false than vice versa when falsity was prioritized but not when truth was prioritized.

In the second experiment value influenced only context recollection in memory for truth and falsity, although in the first experiment we also observed better target recollection for the high-value items. This inconsistency may be due to differences in the design between experiments and the fact that the context was determined based on different information in Experiments 1 and 2 – due to the structure of the model, we could only ask participants about one type of context at a time.

Previously, we found that context recollection for true sentences was significantly decreased due to cognitive load (Ford & Nieznański, 2023), so we inferred that more cognitive resources are allocated to processing of true sentences. Specifically, we showed that rehearsal interference lowered context recollection for truth and falsity similarly, so that the advantage of truth memory over falsity observed for the no-load condition persisted. In contrast, refreshing interference more selectively lowered context recollection for truth, eliminating the truth memory advantage. Since refreshing is engaged in semantic processing (Abadie & Camos, 2019), these results suggest that deep encoding is more involved in processing true sentences than false ones. In a similar vein, Elliott and Brewer (2019) showed that cognitive load attenuates the effect of value on memory, but only when the distracting task was difficult and engaged executive resources. In the case of articulatory rehearsal distracting task, which interferes with shallow processing, the value effect was not diminished. In conclusion, both assigning value to an item and assigning a truth label to it lead to improved remembering, which can be eliminated by impairing elaborative processing, but not shallow rehearsal. For both true and important (but not false and unimportant) information participants may be motivated to assimilate it into their knowledge. Such incorporation into existing knowledge boosts memory, but requires a deeper level of processing. Alternatively, both true (important) and false (unimportant) information is automatically assimilated into knowledge, but false information is actively inhibited, and this inhibition process requires executive resources. In this scenario, the difficult distraction task condition would lead to an increase in memory for false sentences compared to the no-load condition, as we observed in our earlier experiment (Ford & Nieznański, 2023) for target recollection, but not for context recollection.

It seems that inhibition may be involved in the processing of false information, which would explain why prioritizing it is not efficient in case of falsity. The inhibition process occurs when statements are negated (Beltrán et al., 2019), and it is possible that when presented with falsity, people simply withdraw their cognitive resources from processing it. False information could even be perceived as aversive, contaminating our knowledge, and aversive events mostly block the dopamine response (Schultz, 2013). Conversely, value-directed remembering involves reward and activates dopaminergic brain regions (for review, see Knowlton & Castel, 2022), so adding a high value to false information may simply invoke conflicting effects, which cancel each other out.

A limitation of the second experiment was that we did not have a control condition with no value provided, so comparisons with the “default” levels of processing could not be analyzed. If we had had a control condition, we could have offered some suggestions as to the additive nature of the effects of prioritization and veracity on memory, but we can only refer to it based on the context recollection parameter for true sentences in the true-prioritized condition and compare it to the false-prioritized condition. However, this comparison seems to be insufficient, and a reliable evaluation of the additive effect is not possible because of the lack of a control condition. Therefore, more research on the mechanisms underlying memory for true information versus important information is needed. Additionally, we manipulated the prioritization of the sentences in two separate conditions, and it would be interesting to investigate interactions between factors when both are manipulated within subjects.

In most studies on prioritization, authors used single words (Villaseñor et al., 2021), or pictures (Jeanneret et al., 2023) as materials. In our study, we introduced trivia sentences to this field of research, which is a relatively novel direction in the VDR research (e.g., Chung & Federmeier, 2023). Trivia sentences are meaningful, complex, and can be consolidated with the subject’s knowledge. This paper also introduces a new direction in the research on memory for truth and falsity. Value has never been considered as a contributing factor in the research on memory for truth and falsity. This can be due to the fact that typically prioritization procedures have been used in working memory studies, and there have been inconsistent findings in the value effects on long-term memory (see Jeanneret et al., 2023). The presented experiments show clearly that there is a boost in long-term memory provided through value-directed remembering. Additionally, it shows that value is a contributing factor in encoding true and false information. The exact mechanisms of the process require further examination.

## Declarations

### Open practices statement

The data, materials and code for the experiments are available at https://osf.io/9gzyc/. None of the experiments were preregistered.
